# Kimura’s disease mimicking thoracic spine dumbbell neurogenic tumor: a case report and literature review

**DOI:** 10.1186/s12893-020-00870-0

**Published:** 2020-09-21

**Authors:** Siwei Bi, Jun Gu, Chenggong Hu

**Affiliations:** 1 West China School of Medicine, Sichuan University, Chengdu, China; 2 Department of Cardiovascular Surgery, West China Hospital of Sichuan University, Leshan, China; 3 Department of Critical Care Medicine, West China Hospital of Sichuan University, No 37 Guo Xue Xiang, Chengdu, Sichuan 610041 People’s Republic of China

**Keywords:** Kimura’s disease, Eosinophilia, Immunoglobulin E, Lymphadenopathy, Thoracic spine dumbbell tumor

## Abstract

**Background:**

Kimura’s disease is a rare, benign chronic inflammatory disease of unknown etiology that mostly affects Asians. The disease typically presents as subcutaneous masses in the head or neck region that are predominantly found in the preauricular and submandibular areas.

**Case presentation:**

A 7-year-old boy presenting with paralysis of both lower extremities and a thoracic spine dumbbell mass was initially diagnosed with a neurogenic tumor, but the pathological and laboratory examinations confirmed the diagnosis of Kimura’s disease. The paralysis symptom disappeared rapidly, but the patient had developed a recurrent mass in the cervical vertebral canal at the 9-month follow-up.

**Conclusion:**

To our knowledge, no prior published literature has revealed Kimura’s disease cases that mimic dumbbell neurogenic tumors. Here, we report such a case of Kimura’s disease for the first time and provide a brief review of the literature.

## Background

Kimura’s disease is a rare chronic inflammatory disorder that was first reported in China by Kim and Szeto in 1937 [1] and became more widely known after a systematic description was published in 1948 by Kimura et al. [2]. Kimura’s disease mainly affects young Asian (Chinese and Japanese) men between 20 and 40 years of age, although sporadic cases have been described elsewhere [3–5]. Clinically, it typically presents as nontender subcutaneous single or multiple nodules in the head and neck regions, which are predominantly found in the preauricular and submandibular area. Masses in the orbit [6, 7], eyelid [8], epiglottis [9], earlobe [10], lacrimal gland [11], parotid gland [12, 13], groin [14], breast [15], and long bones [16] have also been reported. However, to our knowledge, there have been no reports of Kimura’s disease presenting as a posterior mediastinal dumbbell mass extending into the vertebral canal through the intervertebral foramen.

## Case presentation

**Fig. 1:**
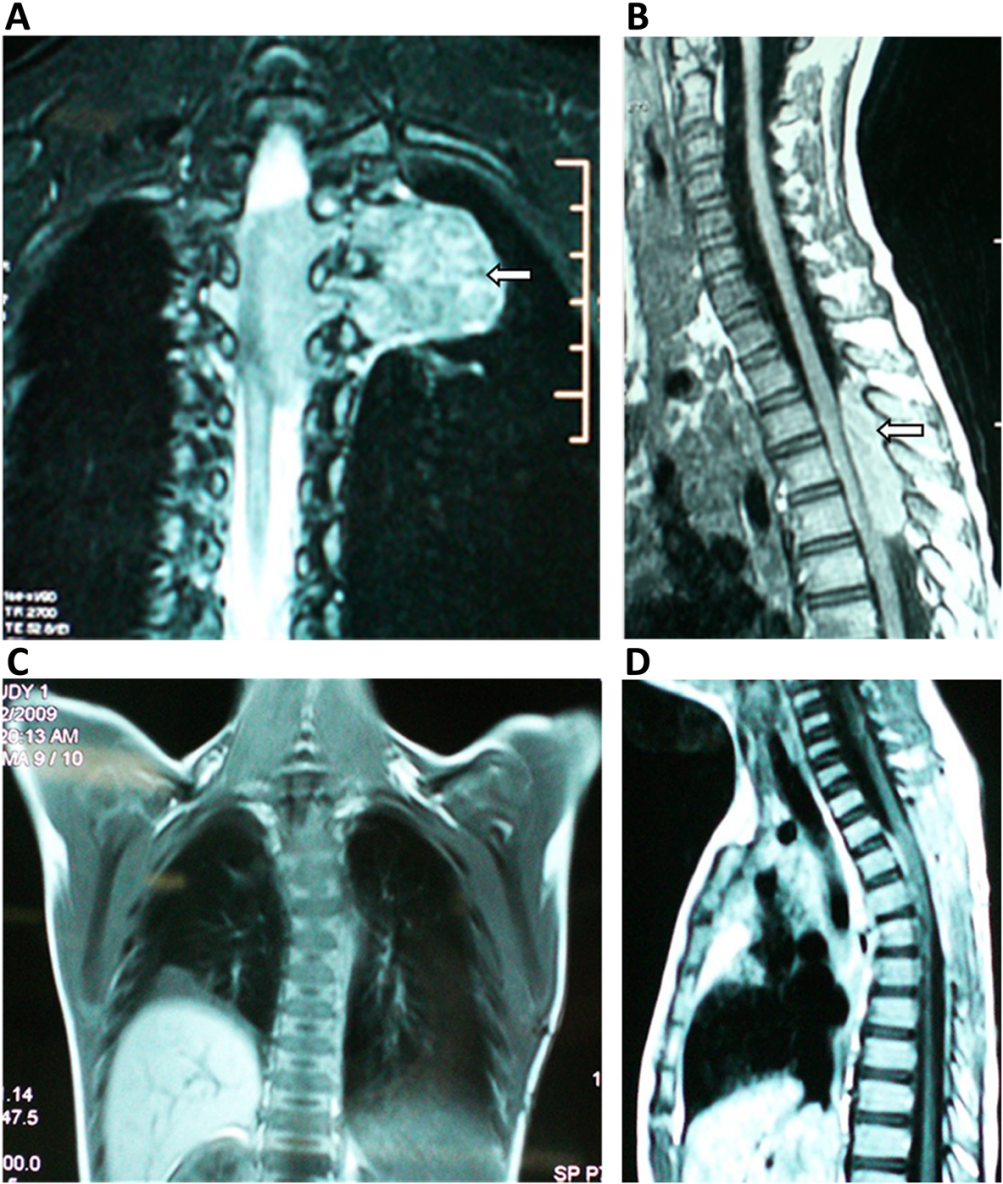
Magnetic resonance imaging (MRI) before (**a**, **b**) and after treatment (**c**, **d**). **a** the chest coronal MRI showed a dumbbell-shaped mass (arrow) extending to the spinal canal and paravertebrally through an enlarged intervertebral foramen; **b** sagittal MRI indicated that the mass was located in the spinal canal between T3 and T5 (arrow) and measured up to 5 cm in length. The spinal cord was compressed and displaced; **c** coronal MRI showed that there was no mass found in the thoracic cavity; **d** sagittal MRI showed that there was no mass found in the spinal canal between T3 and T5

A 7-year-old boy was admitted to our hospital on April 2, 2018, with a complaint of paralysis in both lower extremities lasting for 4 days. Physical examination revealed that he could not move his lower extremities or control urination and defecation. His tendon reflex had disappeared completely in the lower extremities. Some enlarged lymph nodes were found in the neck region. The chest coronal magnetic resonance imaging (MRI) showed a dumbbell-shaped mass in the thoracic cavity between T3 and T5 that measured up to 5 cm in diameter (Fig. 1a). Horizontal MRI indicated that the mass extended to the spinal canal and paravertebral region through an enlarged intervertebral foramen. The spinal cord was compressed and obviously displaced (Fig. 1b). The mass was considered an extradural and paravertebral dumbbell-shaped neurilemmoma. On April 3, 2018, the patient underwent surgery for excision of the lesions using a posterior approach. Under general anesthesia, he was intubated with a double-lumen endotracheal tube and was placed in the right semilateral position. Initially, laminectomy was performed from the lower T3 to the upper T5 by making a vertical linear skin incision from T3-T5. An encapsulated yellowish tumor attached to the dura mater was observed through the left intervertebral foramen between T3 and T4. The mass was connected to the root of the third intercostal nerve, which was ligated and sheared. Subsequently, the chest surgeon induced the collapse of the lung and inserted a thoracoscope through the fourth left intercostal space of the clavicular midline. The mass was observed to protrude from the parietal pleura of the third left intercostal space. Therefore, two thoracic portals were added along the fifth intercostal space of the anterior chest wall. Under the thoracoscope, we separated the mass along with the capsule, carefully confirming the sympathetic nerve. As a result, the mass partially crumbled, but we were able to extract it from the pleural cavity. A chest tube was placed direct under vision, the lung was re-expanded, and the other three portals were closed. The duration of the operation was 3 h.

**Fig. 2:**
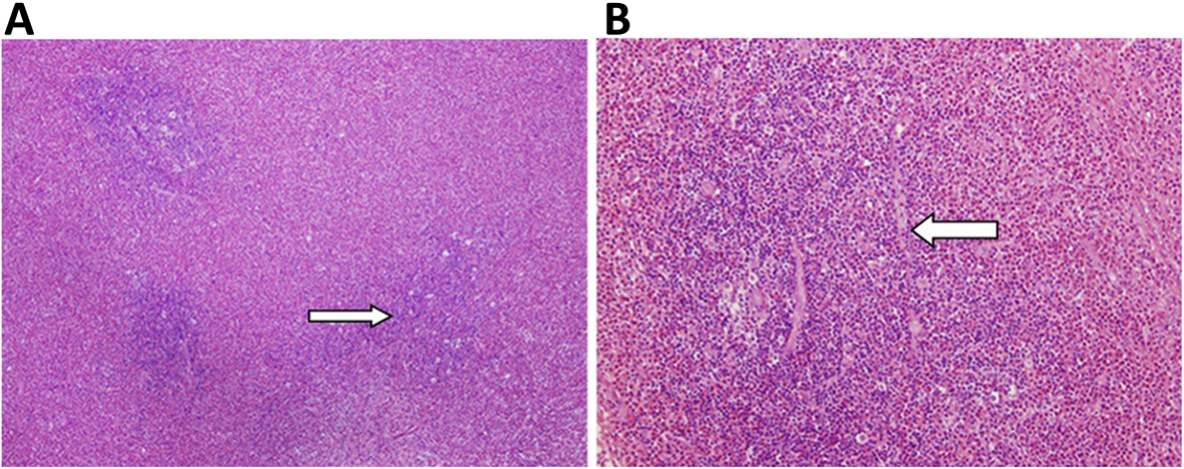
Histopathological examination of the excised tumor. **a** The mass showed prominent infiltration by eosinophils with formation of eosinophilic micro abscesses and hyperplasia of germinal centers (arrow); **b** there was massive infiltration by eosinophils, predominantly with eosinophilic aggregation in some areas (arrow)

**Fig. 3:**
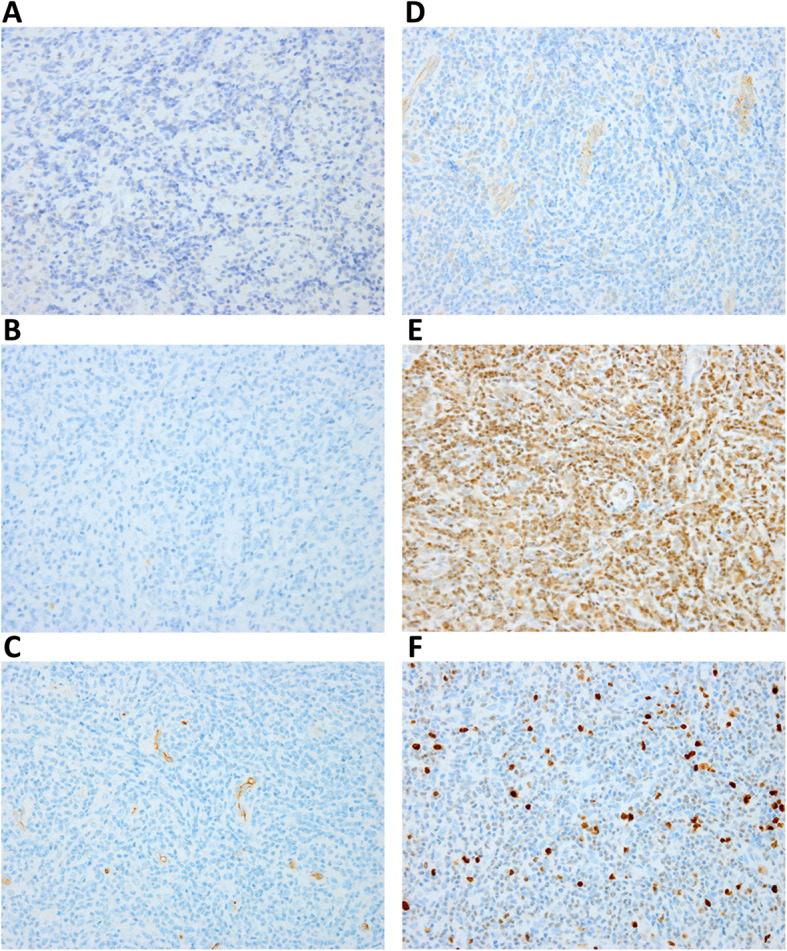
Immunohistological staining of CD1a (**a**), S-100 (**b**), CD34 (**c**), CD31 (**d**), Fli (**e**) and Ki-67 (**f**) (400×)

Histopathological examination of the excised tumor revealed numerous lymphoid follicles with hyperplastic germinal centers. There was massive and prominent infiltration of eosinophils with a few areas that were occupied by eosinophilic microabscesses (Fig. 2), which indicates the eosinophilic hyperplastic lymphogranuloma (Kimura’s disease). The results of laboratory examination were obtained after the operation due to the rapid progression of neurologic deficit and showed that the red blood cell count was 4.09 × 1012/L, hemoglobin was 111 g/L, the white blood cell count was 13.77 × 109/L, platelets were 280 × 109/L and the absolute eosinophil count was 5.78 × 109/L, and there was 42% eosinophilia. Serum immunoglobulin E (IgE) was increased to 572 IU/mL (normal < 250). The other results, including blood urea nitrogen (6.83 mmol/L), serum creatinine level (53.2 μmol/L), and urinalysis, were normal. Immunohistological staining (Fig. 3) was later performed, showing negative staining for CD1a, S-100, and CD34 and positive staining for CD31, Fli and Ki-67. These results confirmed the diagnosis of Kimura’s disease.

The patient, therefore, was started on 40 mg/day prednisone and responded well after 1 week. The eosinophilia and IgE were stabilized with 5 mg of prednisolone. Two weeks later, the patient could move his lower extremities in the bed. One month later, he could walk with his mother’s help. At the 6-month follow-up, the patient was symptom-free and did not demonstrate any sign of recurrence (Fig. 1c, d). At the 9-month follow-up, the patient had developed a recurrent mass in the cervical vertebral canal with the tapering of medication. However, the patient refused further treatment, and further information is not available.

## Discussion and conclusions

We report the case of a 7-year-old boy who complained of paralysis in both lower extremities who had a dumbbell mass in the postmediastinum after MRI examination. The clinical picture initially indicated a neurogenic tumor. Biopsy and histological examination, however, finally identified Kimura’s disease.

Histopathologically, the mass associated with Kimura’s disease is usually characterized by the formation of multiple lymphoid follicles with prominent germinal centers, many of which are infiltrated by eosinophils. Eosinophilic infiltration is massive, with the formation of eosinophilic abscesses [17, 18]. This feature can distinguish Kimura’s disease from angiolymphoid hyperplasia with eosinophilia (ALHE), in which lymphoid infiltration is more diffuse and lymphoid follicles and eosinophilic abscesses are only occasionally observed [17, 18]. In addition, in contrast to those in patients with ALHE, peripheral blood eosinophil counts and serum IgE levels are markedly elevated in patients with Kimura’s disease, which was also found in our case. Nephrotic syndrome is also a common presentation, occurring in up to 60% of patients [19]; however, it was not observed in our case. Our patient had normal levels of urea and creatinine and normal urinalysis results. Few studies have focused on the immunohistochemical examination of tissues in patients with Kimura’s disease. Birol et al. showed the positive expression of CD68, CD34, leukocyte common antigen (LCA) and S-100 [20]. Sun et al. reported the presence of LCA, vimentin (VIM), S-100, CD3, CD45RO, CD20, CD79a, CD31, CD34, F8, c-Kit, and platelet-derived growth factor receptor (PDGFR)-α in Kimura’s disease [21]. However, our results revealed positivity for CD31, Fli and Ki-67 but negativity for CD1a, S-100, and CD34, which were chosen to exclude Langerhans cell granulomatosis [22]. Tumors were considered to originate from Langerhans cells when the neoplastic cells expressed CD1a and S-100 [23].

Therapies for Kimura’s disease include surgical excision, steroids, radiation, and immunosuppressive agents (e.g., cyclosporine). Although they can reduce the size of the lesion and delay disease progression, recurrence is common [24, 25]. In the present case, the patient was treated with a combination of resection of the lesion and oral steroids. Although the patient’s clinical symptoms improved remarkably immediately after the surgery, the patient developed a recurrent mass in the cervical vertebral canal after a 9-month follow-up since the tapering of medication. We planned to prepare for another surgery, radiotherapy, and cyclosporine treatment for the patient, but his parents refused further treatments owing to financial difficulty. Through our search of the PubMed database, we summarized all recurrent Kimura’s cases (Table 1). Notably, there were no neurologic syndrome noted in all the previously published recurrent Kimura’s cases and all the reported reasons for recurrent were tapering of medication.

**Table 1:** Clinical features, treatments, and outcomes of recurrent cases with Kimura Disease

Authors	No. of patients	Year	Country	Male/Female	Age at onset (year)	Size (cm)	Location	Treatment	Nephrotic Syndrome	Blood eosinophil (%)	Serum IgE (IU/mL)	Recurrence	Reason for recurrence	Follow-up duration (month)
Kung, I. T. [26]	21	1984	/	18 Male/ 3 Female	7–50	3–10 in diameter	head, neck, groin, upper limb and chest wall	surgical excision	no	12%; 30%; others not clear	/	5 recurrent cases	/	not clear
Chow, L. T. [27]	8	1994	/	7 Male/ 1 Female	9–70	0.9 × 1.5; 1.0 × 2.0; 1.0 × 1.5; 3.0 × 2.5; 3.0 × 2.5; 1.0 × 1.0 × 3.0 others not clear	head and neck	surgical excision, radiation therapy	no	/	/	2 recurrent cases	/	6–48
Armstrong, W. B. [28]	2	1998	Vietnamese	2 Male	14; 48	1 × 2, 2 × 3 to 6 × 4; 5 × 7	head and neck	prednisone, surgical excision	yes (1)	7.9 to 13%; 22%	/	2 recurrent cases	/	6; not clear
Tsukadaira, A. [29]	1	1998	/	Male	70	8 × 5, 1 × 2	groin, buttock, brachium, neck, popliteal	surgical excision	no	8040/mL	16,700	recurrent	/	not clear
Gumbs, M. A. [30]	1	1999	/	Female	55	12 cm in diameter	head	surgical excision	yes	45%	/	recurrent	/	180
Okami, K. [31]	1	2003	Japanese	Male	14	/	neck	CO2 laser excision, prednisolone of 30 mg	no	16.4%	1260	recurrent	/	12
Chen, H. [32]	21	2004	7 Caucasians, 6 Blacks, 6 Asians, 1 Hispanic, and 1 Arabic	18 Male/ 3 Female	8–64	1.2–6.5	posterior auricular, cervical, groin, and epitrochlear region	surgical excision, corticosteroid therapy, radiation therapy	no	/	/	5 recurrent cases	/	14.4–399.6
Birol, A. [20]	1	2005	Caucasian	Male	45	3.4 × 2.5, 2.6 × 1.5, 4.2 × 3.5	head	steroid, cyclosporine 5 mg/kg/day	no	36%	1130	recurrent	tapering of steroid or cyclosporine	5
Chitapanarux, I. [33]	8	2007	/	6 Male/ 2 Female	24–54	/	head and neck	surgical excision, radiation therapy	no	/	/	8 recurrent cases	/	21–43
Kilciksiz, S. [34]	1	2007	/	Male	32	5 × 5	neck	surgical excision, prednisolone 1 mg/kg/day, radiation therapy	no	6%	242	recurrent	/	31
Meningaud, J. P. [35]	2	2007	Madagascar, Mauritius native	Male	29; 25	/, 8.5 × 3.5	head	surgical excision	no	/	/	1 recurrent case	/	12; 12
Shin, S. T. [36]	1	2007	/	Male	8	/	head, arm and axillary region	surgical excision, steroid 60 mg/day, cyclosporine-A 2 mg/kg/day, azathioprine 1.5 mg/kg/day	no	21%	> 2000	recurrent	/	17
Wang, D. Y. [37]	1	2009	Chinese	Male	6	1.5 × 1.5	neck	25 mg of prednisone	yes	32.0%	> 400	recurrent	/	not clear
Soeria-Atmadja, S. [38]	2	2011	Philippine, Bangladesh	2 Male	17; 9	4 × 5; 2 × 3	head and neck	prednisolone 1 mg/kg/day, cyclosporine 4 mg/kg/day; prednisolone 2 mg/ kg/ day, cyclosporine	yes	4.4 × 10^9^ / L; 8.3 × 10^9^ / L	5000 kU/L; > 5000 kU/L	recurrent	tapering of prednisolone	9; not clear
Shahryari, J. [24]	1	2012	Irani	Male	45	6 × 4 × 1.5	head	surgical excision	no	23%	100	recurrent	/	not clear
Beccastrini, E. [3]	1	2013	Italian	Male	40	9 in diameter	trunk, elbow, wrist and hip	prednisone 25 mg/day, CSA 3 mg/kg/day	no	1900 /mL	1578 KU/L	recurrent	tapering of CSA	113
Wang, Z. [39]	1	2014	Chinese	Male	53	1.7 × 1.1 × 1.1	neck	surgical excision	no	1.01 × 10^9^ / L	537.2	recurrent	/	68
Hsu, S. N. [40]	1	2015	Chinese	Male	33	/	head, lower extremity (edema)	surgical excision, PTA, Cilostazol 50 mg, Pentoxifylline 400 mg, prednisolone 1 mg/kg/day, Cetirizine 5 mg twice a day	no	34 to 51%	12,400-17,200	recurrent	/	not clear
Ye, X. [41]	1	2015	Chinese	Male	47	5 in diameter	cervical, subaxillary and inguinal region	prednisone 0.5 mg/kg/day, thalidomide 50 mg/day	no	26.11%	1081.34	recurrent	tapering of prednisolone	36
Wang, H. [42]	1	2016	Chinese	Male	72	/	head	surgical excision, Chinese herbal remedies, cetirizine hydrochloride and olmesartan 20 mg/day, gamma immunoglobulin 10 g/day for 5 days, intravenous pulse methylprednisolone therapy 500 mg for 3 days, hydroxychloroquine 0.4 g/day, prednisone 50 mg/day, a single dose of intravenous cyclophosphamide 500 mg	yes	35%	149,000	recurrent	/	43
Matsuo, T. [11]	1	2017	Japanese	Male	42	/	head	surgical excision, prednisone 40 mg/day, cyclosporine 75 mg/day	yes	9.40%	735	recurrent	tapering of prednisolone	82
Chakraborti, C. [43]	1	2019	/	Female	23	2.5 × 2.5 × 1	head and neck	prednisolone 40 mg	no	30%	262.64	recurrent	/	2
Li, X. [44]	1	2019	Chinese	Male	48	15 × 10 × 3, 5 × 5 × 2, 4 × 3 × 2, 4 × 3 × 1	head and neck	surgical excision, 25 mg prednisone twice daily	no	0.55%	27,100	recurrent	/	0.5
Zhang, G. [45]	24	2020	/	20 Male/ 4 Female	5–65	/	head and neck	surgical resection, radiotherapy, oral corticosteroids	no	11 to 51%	/	11 recurrent cases	/	6–113
Siwei, B.(current)	1	2020	Chinese	Male	7	5	thoracic cavity	prednisone 40 mg/day	no	42%	572	recurrent	tapering of prednisolone	9

*CSA* Cyclosporine A, *PTA* Percutaneous transluminal angioplasty

In conclusion, we reported our experience managing a rare case of Kimura’s disease presenting as a posterior mediastinal dumbbell mass. Although the short-term outcome was good, the patient experienced recurrence at 9 months after surgery. Therefore, additional studies are still warranted to develop an optimal management regimen for rare disease entities.

## Data Availability

All data supporting the conclusions of this study are included in this published article.

## Abbreviations

VIM: Vimentin
PDGFR: Platelet-derived growth factor receptor
LCA: Leukocyte common antigen
ALHE: Angiolymphoid hyperplasia with eosinophilia
MRI: Magnetic resonance imaging
IgE: Immunoglobulin E
